# Antineoplastic effects of selective CDK9 inhibition with atuveciclib on cancer stem-like cells in triple-negative breast cancer

**DOI:** 10.18632/oncotarget.26468

**Published:** 2018-12-18

**Authors:** Daphne Brisard, Frank Eckerdt, Lindsey A. Marsh, Gavin T. Blyth, Sarika Jain, Massimo Cristofanilli, Dai Horiuchi, Leonidas C. Platanias

**Affiliations:** ^1^ Robert H. Lurie Comprehensive Cancer Center of Northwestern University, Chicago, Illinois, USA; ^2^ Department of Pharmacology, Feinberg School of Medicine, Northwestern University, Chicago, Illinois, USA; ^3^ Department of Neurological Surgery, Feinberg School of Medicine, Northwestern University, Chicago, Illinois, USA; ^4^ Division of Hematology/Oncology, Department of Medicine, Feinberg School of Medicine, Northwestern University, Chicago, Illinois, USA; ^5^ Department of Medicine, Jesse Brown VA Medical Center, Chicago, Illinois, USA

**Keywords:** triple-negative breast cancer (TNBC), cancer stem-like cells, CDK9, MYC, atuveciclib

## Abstract

Treatment options for triple-negative breast cancer (TNBC) are limited due to the lack of efficient targeted therapies, frequently resulting in recurrence and metastatic disease. Accumulating evidence suggests that a small population of cancer stem-like cells (CSLCs) is responsible for tumor recurrence and therapy resistance. Here we investigated the role of cyclin-dependent kinase 9 (CDK9) in TNBC. Using The Cancer Genome Atlas (TCGA) data we found high-*CDK9* expression correlates with worse overall survival in TNBC patients. Pharmacologic inhibition of CDK9 with atuveciclib in high-*CDK9* expressing TNBC cell lines reduced expression of CDK9 targets *MYC* and *MCL1* and decreased cell proliferation and survival. Importantly, atuveciclib inhibited the growth of mammospheres and reduced the percentage of CD24^low^/CD44^high^ cells, indicating disruption of breast CSLCs (BCSLCs). Furthermore, atuveciclib impaired 3D invasion of tumorspheres suggesting inhibition of both invasion and metastatic potential. Finally, atuveciclib enhanced the antineoplastic effects of Cisplatin and promoted inhibitory effects on BCSLCs grown as mammospheres. Together, these findings suggest CDK9 as a potential therapeutic target in aggressive forms of *CDK9*-high TNBC.

## INTRODUCTION

Breast tumor subtypes are defined based on the expression of three primary identifiers: estrogen receptor (ER), progesterone receptor (PR) and human epidermal growth factor receptor 2 (HER2). Breast tumors lacking all three markers are referred to as triple-negative breast cancer (TNBC), which account for 15–20% of all breast cancer cases [1, 2]. Treatment options for patients with TNBC are currently limited to the combinations of conventional chemotherapy in both primary and metastatic setting, radiation, and surgery in early disease [1, 2]. TNBC frequently develops early recurrence after initial treatment and displays a tendency to metastasize primarily to visceral sites such as the lung, liver and brain resulting in significantly reduced overall survival [3, 4]. Metastatic TNBC represents one of the most clinically challenging conditions in patients with breast cancer [5, 6]. Evidence indicates that metastasis in various cancer types including breast cancer is promoted by a small subset of tumor cells termed cancer stem-like cells (CSLCs) [7]. These cells are pluripotent, able to self-renew, and eventually may give rise to malignant tumors [8, 9]. CSLCs also mediate resistance to the current standard of care including chemotherapy and radiation, resulting in poor clinical outcomes [7, 10].

Breast cancer stem-like cells (BCSLCs) are currently identified based on protein expression levels of cell-surface markers such as high CD44 and low CD24, and enzymatic activity of aldehyde dehydrogenase (ALDH1) [11, 12]. The CD44^high^/CD24^low^ population has been shown to have an increased ability to undergo epithelial-mesenchymal transition, thereby rendering tumor cells more motile and migratory, which is critical to the initiation of breast cancer metastasis [13]. Metastatic TNBC is currently considered incurable [1, 5, 6], underscoring the need for new therapeutic strategies. Data from patient-derived tumor xenograft (PDX) models have suggested important roles for CDKs in the metastatic phenotype and in preclinical studies the pan-CDK inhibitor dinaciclib demonstrated antineoplastic activity [14]. This suggests important roles for CDKs in breast cancer invasion and metastasis, but, the precise contribution of each CDK in TNBC tumorigenesis remains to be defined.

CDKs are frequently dysregulated in human malignancies, and thus their contributions to tumorigenesis and tumor maintenance have been extensively studied [15]. CDKs have long been considered as potentially promising therapeutic targets, and several CDK inhibitors with different CDK specificities have been evaluated in clinical trials [16, 17]. In hormone receptor positive metastatic breast cancer pan-CDK inhibitors (e.g., CDK4/6) demonstrated significant clinical activity and have been FDA-approved for the management of both endocrine-sensitive and resistant disease irrespective of menopausal status [18–24]. However, pan-CDK inhibitors may cause dose-limiting toxicity (e.g. neutropenia as class effect and GI toxicity with abemaciclib) and this has limited combination with other cytotoxic agents and investigations in TNBC [18–24]. CDK9 interacts with T-type cyclins to form the positive transcription elongation factor b (P-TEFb) [25]. P-TEFb is involved in mRNA processing through phosphorylation of serine (Ser)-2 residue of the carboxyl-terminal domain of the large subunit of RNA polymerase II (RNA Pol II) to promote elongation of pro-tumorigenic factors including MYC and MCL1 [25, 26]. Elevated expression and transcriptional activity of the MYC oncoprotein have been observed in many human malignancies including TNBC [27]. So far, strategies targeting MYC directly have shown limited success [28]. Thus, targeting gene expression of MYC and other pro-tumorigenic factors by CDK9 inhibition may offer potential therapeutic opportunities.

Atuveciclib is a novel small molecule CDK inhibitor that selectively targets CDK9 [29] that is currently under clinical evaluation in multiple early phase clinical trials that enroll TNBC patients (NCT01938638 and NCT02345382) (clinicaltrials.gov). Atuveciclib has been shown to inhibit RNA polymerase II (Ser2) phosphorylation and downregulate MYC protein expression in hematologic malignancies [29]. In the present study, we sought to evaluate the effects of CDK9 targeting in TNBC and study the mechanisms by which CDK9 inhibition may exert antineoplastic effects. We found that elevated CDK9 expression was associated with decreased progression-free survival in breast cancer patients and a worse overall survival among patients with TNBC. In TNBC cell lines exhibiting elevated CDK9 expression, atuveciclib potently inhibited cell proliferation and induced cell death. Furthermore, CDK9 inhibition impaired the growth of TNBC cells in three-dimensional (3D) mammospheres and enhanced the antineoplastic effects of cisplatin. Thus, our observations suggest that specific and selective inhibition of CDK9 activity may represent a promising tool for targeted therapy for patients with TNBC.

## RESULTS

**Figure 1:**
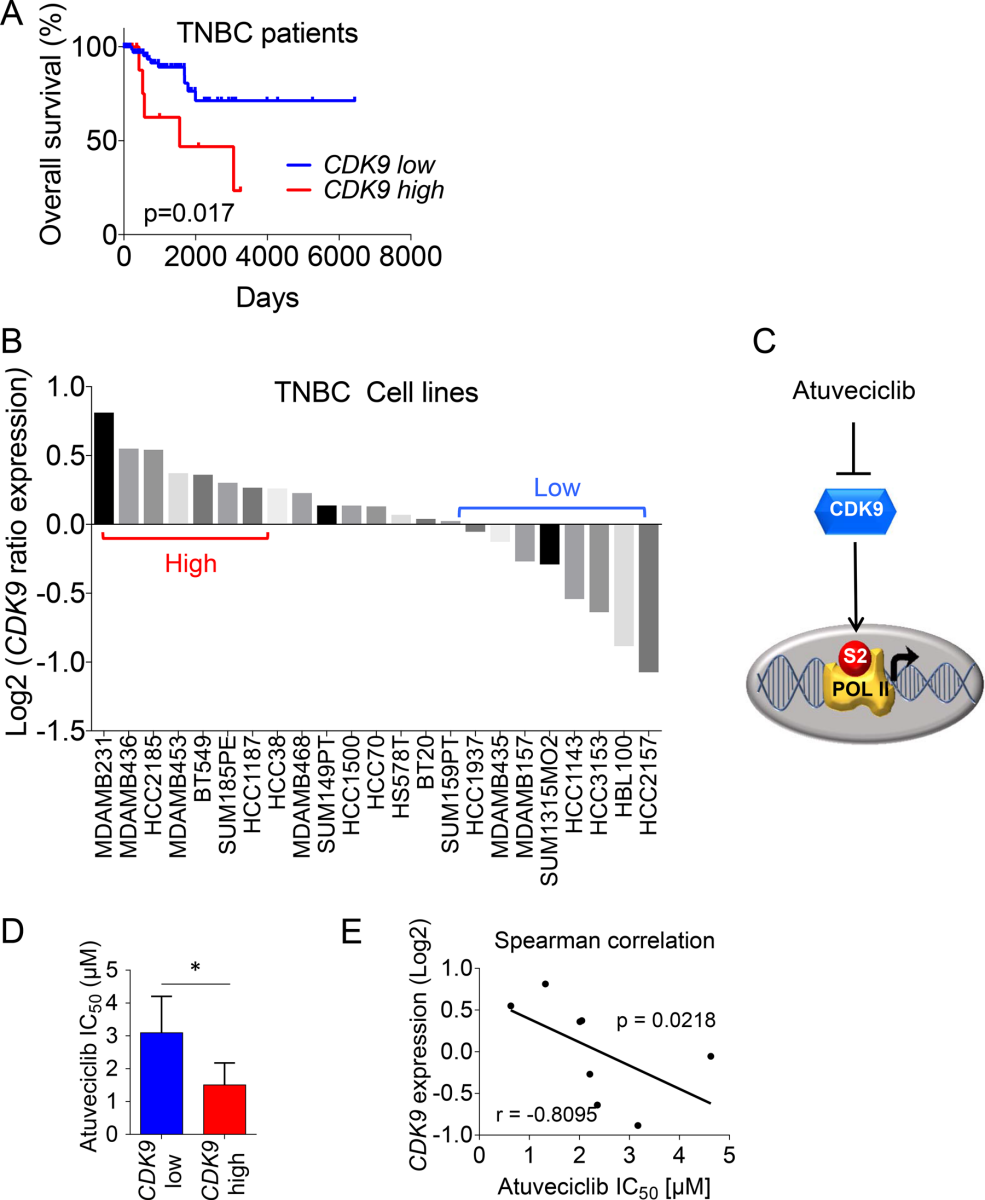
*CDK9* expression in TNBC patients and cell lines (**A**) Kaplan-Meier analysis for overall survival rate based on *CDK9* mRNA expression, RNA-seq data from a TCGA-cohort of 89 TNBC patients is shown. Log-rank (Mantel-Cox), *p* = 0.017. A list of sample IDs is provided in the supplement. (**B**) TNBC cell lines (*n* = 23) from the Neve_2006 dataset were analyzed for *CDK9* expression. Gene expression data from Xena Browser (https://xenabrowser.net/) were ranked using GraphPad Prism. (**C**) Schematic representation of atuveciclib mode of action. (**D**) Comparison of atuveciclib IC_50_ values (see Supplementary Figure 2) between *CDK9*-high (*n* = 4) and – low (*n* = 4) TNBC cell lines. *t*-test was performed. ^*^P < 0.05. (**E**) Log relative *CDK9* expression of TNBC cell lines (*n* = 8), as determined in (B) versus atuveciclib IC_50_ from Supplementary Figure 2. Correlation was assessed using Spearman test (*r* = –0.8095, *P* = 0.0218).

We sought to investigate the relevance of CDK9 targeting in breast cancer cells and the potential therapeutic efficacy of selectively inhibiting CDK9 in TNBC. Gene expression analysis (e.g., RNA-seq) and associated clinical data from the TCGA cohort showed that TNBC patients with tumors exhibiting elevated *CDK9* expression experienced a significantly worse overall survival (OS) (Figure 1A). This effect was not limited to TNBC because high *CDK9* expression was associated with significantly worse relapse-free survival rates in breast cancer patients from an additional independent cohort (Supplementary Figure 1) [30]. These results raised the possibility for a potential contribution of CDK9 to the mechanisms of breast cancer progression. Next, we ranked a panel of TNBC cell lines [31] according to their *CDK9* expression and we were able to group cell lines into high-*CDK9* versus low-*CDK9* cell lines (Figure 1B), suggesting different levels of vulnerability to CDK9 inhibition. To investigate whether CDK9 could be a therapeutic target for patients with TNBC, we sought to explore the effects of the novel small molecule CDK9 inhibitor atuveciclib on TNBC cells. Atuveciclib potently and selectively targets the P-TEFb/CDK9 complex (Figure 1C) [29], thereby inhibiting RNA Pol II function, which is critical for the expression of a number of pro-tumorigenic factors including the MYC oncoprotein [32]. This drug is currently under clinical evaluation in multiple early phase clinical trials (NCT01938638 and NCT02345382) (clinicaltrials.gov). Consistent with the idea of different degrees of CDK9-dependency (see Figure 1B), we found that a panel of high-*CDK9* expressing cell lines (e.g., MDA-MB-231, MDA-MB-436, MDA-MB-453, BT549) exhibited significantly higher sensitivity to atuveciclib as compared to a panel of low-*CDK9* expressing cell lines (e.g., HCC1937, MDA-MB-157, HCC3153, HBL100) (Figure 1D and Supplementary Figure 2). Furthermore, we showed that the atuveciclib IC_50_ values negatively correlated with *CDK9* mRNA expression (Figure 1E), indicating increased sensitivity of high-*CDK9* expressing cell lines to atuveciclib, further suggesting that patients with TNBC that exhibit elevated *CDK9* expression could benefit from a CDK9-targeted therapy.

**Figure 2:**
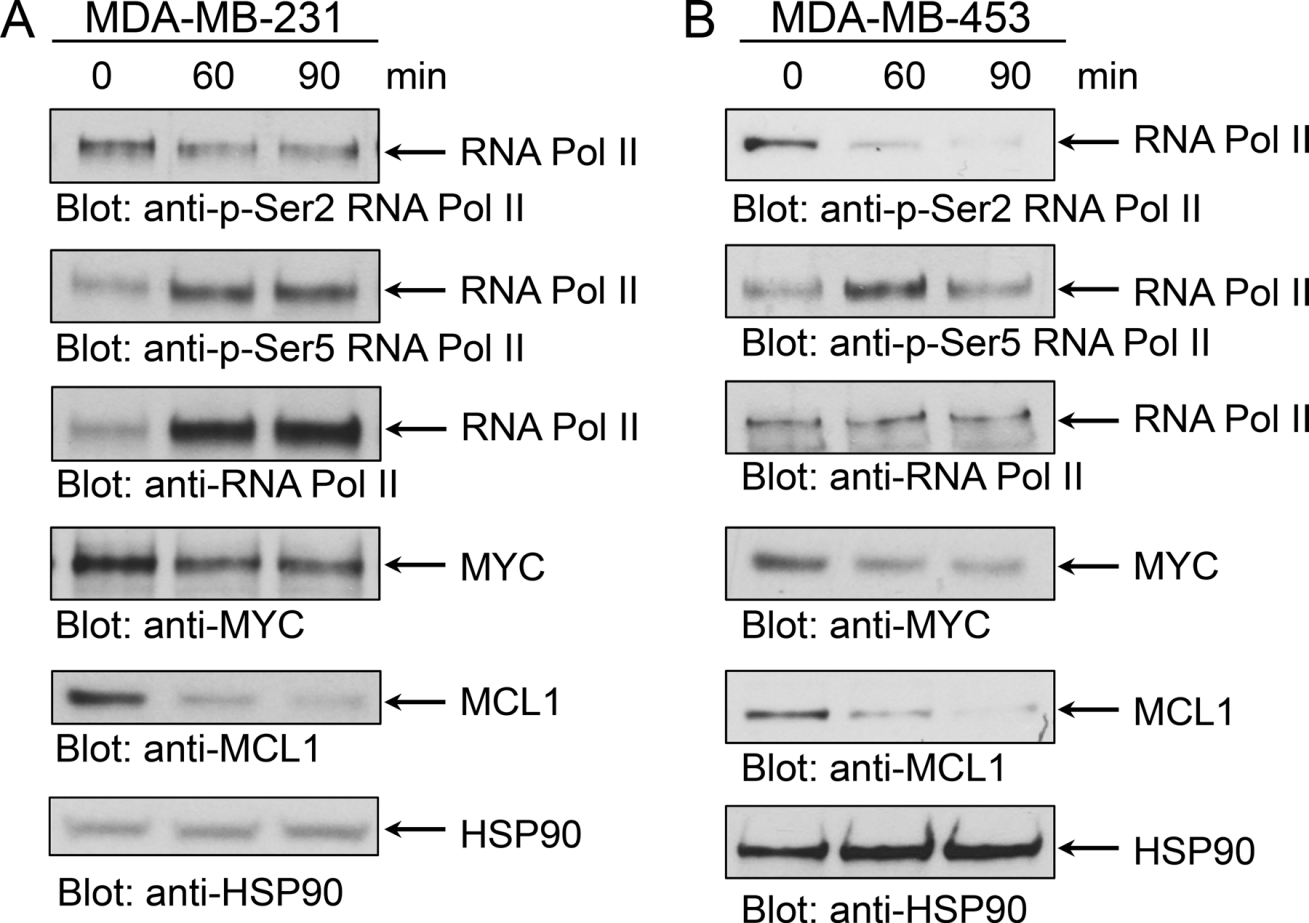
Effect of atuveciclib on RNA Pol II, MYC and MCL1 expression (**A**) MDA-MB-231 cells were treated with atuveciclib (3 μM) for 60 or 90 min. Equal amounts of lysates were analyzed by SDS-PAGE and immunoblotted for antibodies against RNA Pol II pSer2, MYC. Membrane was then stripped and reprobed for antibodies against RNA Pol II. Lysates from the same experiment were analyzed by SDS-PAGE in parallel and immunoblotted for antibodies against RNA Pol II pSer5, MCL1 and HSP90. (**B**) MDA-MB-453 cells were treated with atuveciclib (3 μM) for 60 or 90 min. Equal amounts of lysates were analyzed by SDS-PAGE analysis and immunoblotted for antibodies against RNA Pol II pSer5, MCL1 and HSP90. Membrane was then stripped and reprobed for antibodies against RNA Pol II. Lysates from the same experiment were analyzed by SDS-PAGE in parallel and immunoblotted for antibodies against RNA Pol II pSer2, MYC.

**Figure 3:**
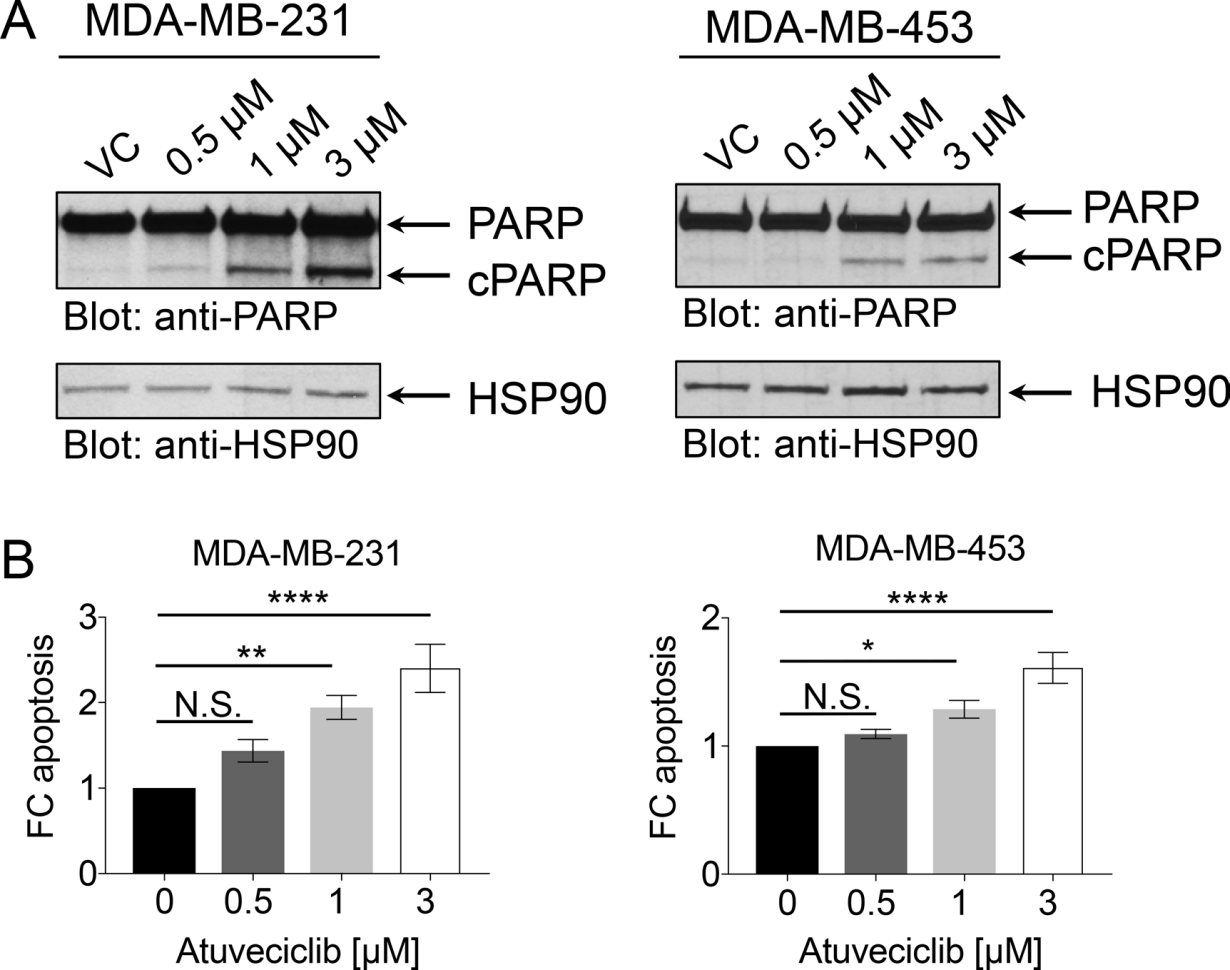
Effect of atuveciclib on apoptosis in TNBC cell lines (**A**) MDA-MB-231 (left panel) or MDA-MB-453 (right panel) cells were treated with vehicle control (VC) or atuveciclib at indicated concentrations for 24 hours. Equal amounts of lysates were analyzed by SDS-PAGE and immunoblotted for antibodies against PARP antibody (which detects both cleaved (cPARP) and full length PARP) and HSP90. (**B**) MDA-MB-231 (left panel) or MDA-MB-453 (right panel) cells were treated with atuveciclib for 4 days at indicated concentrations. Cells were stained with Annexin-V and propidium Iodide (PI) antibodies and then analyzed by flow cytometry. Annexin-V positive and Annexin-V+PI double positive cells were considered apoptotic. Data are presented as the fold change (FC) over VC-treated cells. Results represent the means ± SEM of nine (MDA-MB-231) or six (MDA-MB-453) independent experiments. ^*^P < 0.05; ^**^P < 0.01; ^****^P < 0.0001. N.S. stands for non-significant.

The P-TEFb/CDK9 complex is a key component of transcriptional gene activation, and one of its targets is MYC [25, 26, 33]. MYC is a pleiotropic transcription factor, which has been found overexpressed in TNBC where it is associated with poor clinical outcome [27]. Directly and selectively inhibiting the oncogenic transcriptional activity of MYC using small molecule inhibitors has been challenging primarily due to structural constraints [28]. Atuveciclib has been shown to inhibit MYC expression, which results in anti-tumor activity in multiple xenograft models [29]. Thus, we sought to determine whether atuveciclib reduces MYC expression in TNBC cell lines with high *CDK9* expression. Treatment of TNBC lines with atuveciclib induced rapid, time-dependent dephosphorylation of RNA Pol II on serine 2, an established target for CDK9; whereas it did not result in dephosphorylation of RNA Pol II on serine 5, a target for CDK7 (Figure 2). Second, we found that atuveciclib treatment decreased protein levels of MYC and MCL1 (Figure 2), two major pro-tumorigenic factors regulated by RNA Pol II that are often associated with poor clinical outcomes in many cancer types including TNBC [27, 34]. These results indicate that atuveciclib acts as a potent and specific CDK9 inhibitor in TNBC cell lines. We next examined whether specific CDK9 inhibition with atuveciclib induces cytotoxic effects on TNBC cells. Treatment of high-*CDK9* TNBC cell lines (MDA-MB-231 and MDA-MB-453, see Figure 1B) with atuveciclib induced poly (ADP-ribose) polymerase (PARP) cleavage in a dose-dependent manner (Figure 3A), which was accompanied by a significant increase in Annexin-V and Annexin-V/propidium iodide (PI) positive cells (Figure 3B). By contrast, in the low-*CDK9* cell line HCC1937 (see Figure 1B), atuveciclib only marginally induced PARP cleavage (Supplementary Figure 3A) and the effects on Annexin-V positive cells were less pronounced (Supplementary Figure 3B) as compared to high-*CDK9* cell lines (see Figure 3). Thus high-*CDK9* expressing cell lines are more sensitive to atuveciclib-induced apoptosis as compared to low-*CDK9* expressing cell lines.

**Figure 4:**
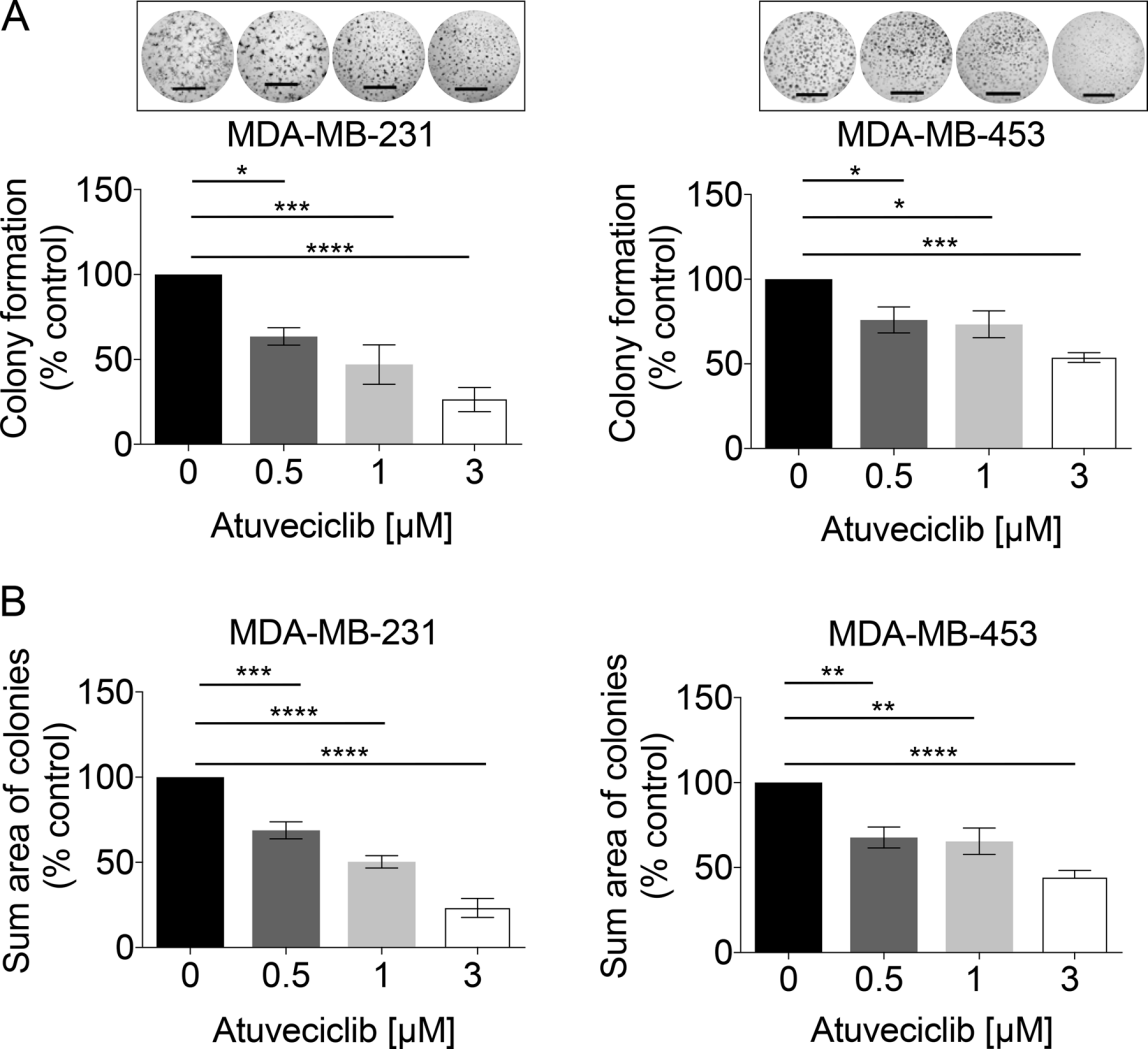
Colony formation analysis in TNBC cell lines in response to increasing doses of atuveciclib (**A**) MDA-MB-231 (left panel) or MDA-MB-453 (right panel) cells were seeded into 96-well plates on a thin layer of EHS tumor extract. After 24 hours, cells were treated with atuveciclib at indicated concentrations. After 6 days entire wells were imaged, and the number of colonies with a diameter ≥ than 60 μm was counted. Data are presented as the percentages of vehicle control (VC) treated cells. Results represent the means ± SEM of four independent experiments for each cell line. Representative images are depicted in the upper panels. ^*^P < 0.05; ^***^P < 0.001, ^****^P < 0.0001. (**B**) MDA-MB-231 (left panel) or MDA-MB-453 (right panel) cells were seeded into 96-well plates on a thin layer of EHS tumor extract. After 24 hours, cells were treated with atuveciclib at indicated concentrations. After 6 days entire wells were imaged, the cross-sectional area of colonies with a diameter ≥ 60 μm was measured and summed up. Sum cross-sectional area data are presented as the percentage of vehicle control (VC) treated cells. Results represent the means ± SEM of four independent experiments for each cell line. Scale bar = 1000 μm. ^**^P < 0.01; ^***^P < 0.001, ^****^P < 0.0001.

Recent evidence suggests 2D monolayer cultures have limited potency for testing drug responses, whereas 3D models appear to better reflect *in vivo* responses [35, 36]. Thus, we employed the Matrigel-assisted 3D-on-top culture method [37] to investigate whether anti-tumor effects of atuveciclib observed in high-*CDK9* TNBC cell lines grown in 2-dimensional (2D) culture could be reproduced in 3D. In high-*CDK9* TNBC cell lines (MDA-MB-231 and MDA-MB-453), atuveciclib markedly reduced the number and size of colonies in a dose-dependent manner (Figure 4); whereas no such anti-tumor effects were observed for a low-*CDK9* TNBC cell line (HCC1937) (Supplementary Figure 4). These observations reinforce the idea that atuveciclib displays anti-tumor effects in TNBC cells with elevated *CDK9* expression in 3D.

**Figure 5:**
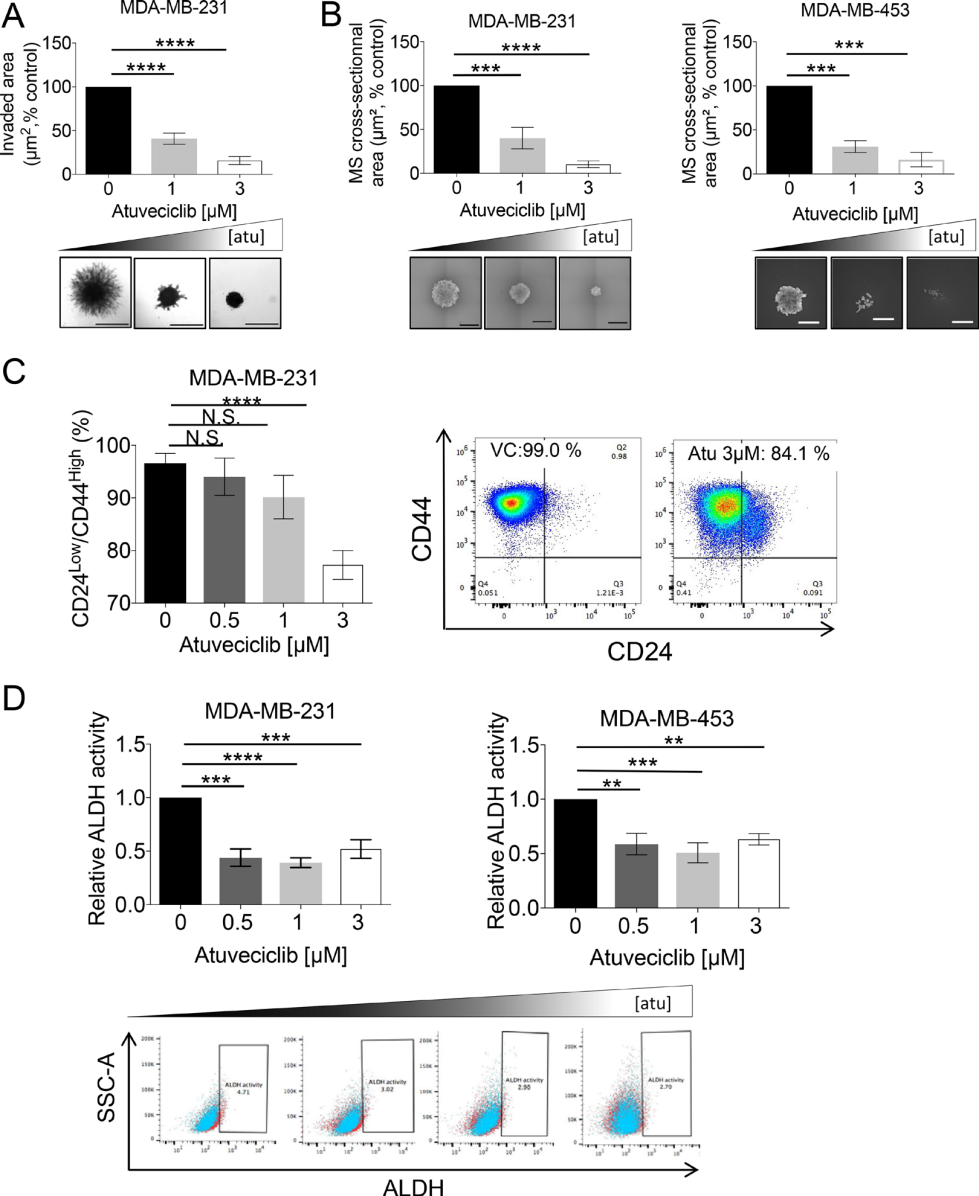
Effect of atuveciclib on mammospheres and stem-like cancer cells from TNBC cell lines (**A**) MDA-MB-231 cells were seeded into 96-well low attachment plates into specialized spheroid formation extra cellular matrix (ECM). After 72 hours, invasion matrix was added and spheres were treated with atuveciclib at indicated concentrations. After 6 days, spheres were imaged and invaded area (μm^2^) was measured with ImageJ software. Data are presented as the percentages of vehicle control (VC) treated cells (upper panel). Results represent the means ± SEM of four independent experiments. Lower panels depict representative images of indicated treatment groups. ^****^P < 0.0001. Scale bar represents 1000 μm. (**B**) MDA-MB-231 (left panel) or MDA-MB453 (right panel) cells were seeded into 96-well low attachment plates to allow formation of mammospheres (MS). Subsequently atuveciclib was added at indicated concentrations. After 8 days, mammospheres were imaged to determine cross-sectional area. Data are presented as the percentages of vehicle control (VC) treated cells. Upper panels: results represent the means ± SEM of four (MDA-MB-231) and three (MDA-MB-453) independent experiments. Lower panels depict representative images of mammospheres from the indicated treatment groups. ^***^P < 0.001, ^****^P < 0.0001. Scale bar represents 1000 μm. (**C**) MDA-MB-231 cells were seeded into 6-well plates and treated with atuveciclib at indicated concentrations. After 4 days, cells were stained with anti-CD24 and anti-CD44 antibodies and analyzed by flow cytometry. Left panel: data are presented as the percentage of CD24^low^/CD44^high^ cells. Results represent the means ± SEM of six independent experiments. Right panels depict representative dot plots of the experiment shown in the left panel. ^****^P < 0.0001. N.S. stands for non-significant. (**D**) MDA-MB-231 (upper left panel) or MDA-MB-453 (upper right panel) cells were seeded into 6-well plates and treated with atuveciclib at indicated concentrations. After 4 days, cells were stained with ALDEFLUOR with or without DEAB followed by flow cytometry analysis. Representative dot plots from the experiment (MDA-MB-231: left, MDA-MB-453: right) are depicted in the lower panels. Data are presented as the fold-change over vehicle control (VC) treated cells. Results represent the means ± SEM of four (MDA-MB-231) and five (MDA-MB-453) independent experiments. ^**^P < 0.01; ^***^P < 0.001, ^****^P < 0.0001.

Our data demonstrating that elevated *CDK9* expression is associated with worse overall survival and with diminished relapse-free survival (Figure 1A and Supplementary Figure 1), indicate a prognostic significance for *CDK9* expression in breast cancer. However, the biological processes by which elevated *CDK9* expression contributes to disease progression remain to be defined. Invasive behavior of tumor cells in their primary sites could facilitate tumor dissemination and thus metastatic progression and CSLCs could play important roles in this process [10]. Using the NKI dataset [38] we found high-*CDK9* expression was associated with significantly worse metastasis-free survival rates in breast cancer patients (Supplementary Figure 5). Thus, we studied the effects of CDK9 inhibition on the invasive phenotype seen in MDA-MB-231 cells and found that atuveciclib significantly inhibited tumorsphere invasion into the surrounding extracellular matrix (ECM) (Figure 5A). This indicates pharmacological CDK9 inhibition may reduce invasive properties of TNBC cells. Motility and migration are often increased in BCSLCs [13], prompting us to investigate the effect of CDK9 inhibition in BCSLCs. To this end, we cultured TNBC cell lines as 3D mammospheres under stem-cell conditions [39]. Interestingly, we found that protein levels of MYC and OCT4, two stem cell/pluripotency markers, were substantially increased in 3D mammospheres as compared with cells grown in 2D (MDA-MB-231 and MDA-MB-453) (Supplementary Figure 6). By contrast, no such increase was observed for CDK9 protein levels (Supplementary Figure 6). However, phosphorylation of RNA pol II on Ser2 markedly increased in 3D mammospheres (Supplementary Figure 6), raising the possibility that cellular demand for CDK9 activity is elevated in cancer stem-like 3D mammospheres. Concomitantly, mammosphere growth was significantly reduced in the presence of atuveciclib in both MDA-MB-231 and MDA-MB-453 cell lines (Figure 5B). Furthermore, we found that the population of CSLCs, identified on the basis of CD44^high^/CD24^low^ expression and ALDH activity, was significantly diminished in these TNBC cell lines following treatment with atuveciclib (Figure 5C and 5D); whereas no such inhibitory effect was observed in HCC1937 cells (Supplementary Figure 7), a low *CDK9*-expressing cell line. These results indicate that atuveciclib-mediated inhibition of CDK9 activity may disrupt CSLCs present in high-*CDK9* TNBC tumors. Together these observations suggest that CDK9 inhibition might be effective in high *CDK9*-expressing TNBCs as it exhibits antineoplastic effects in tumor cells and additionally disrupts tumor-initiating CSLCs.

**Figure 6:**
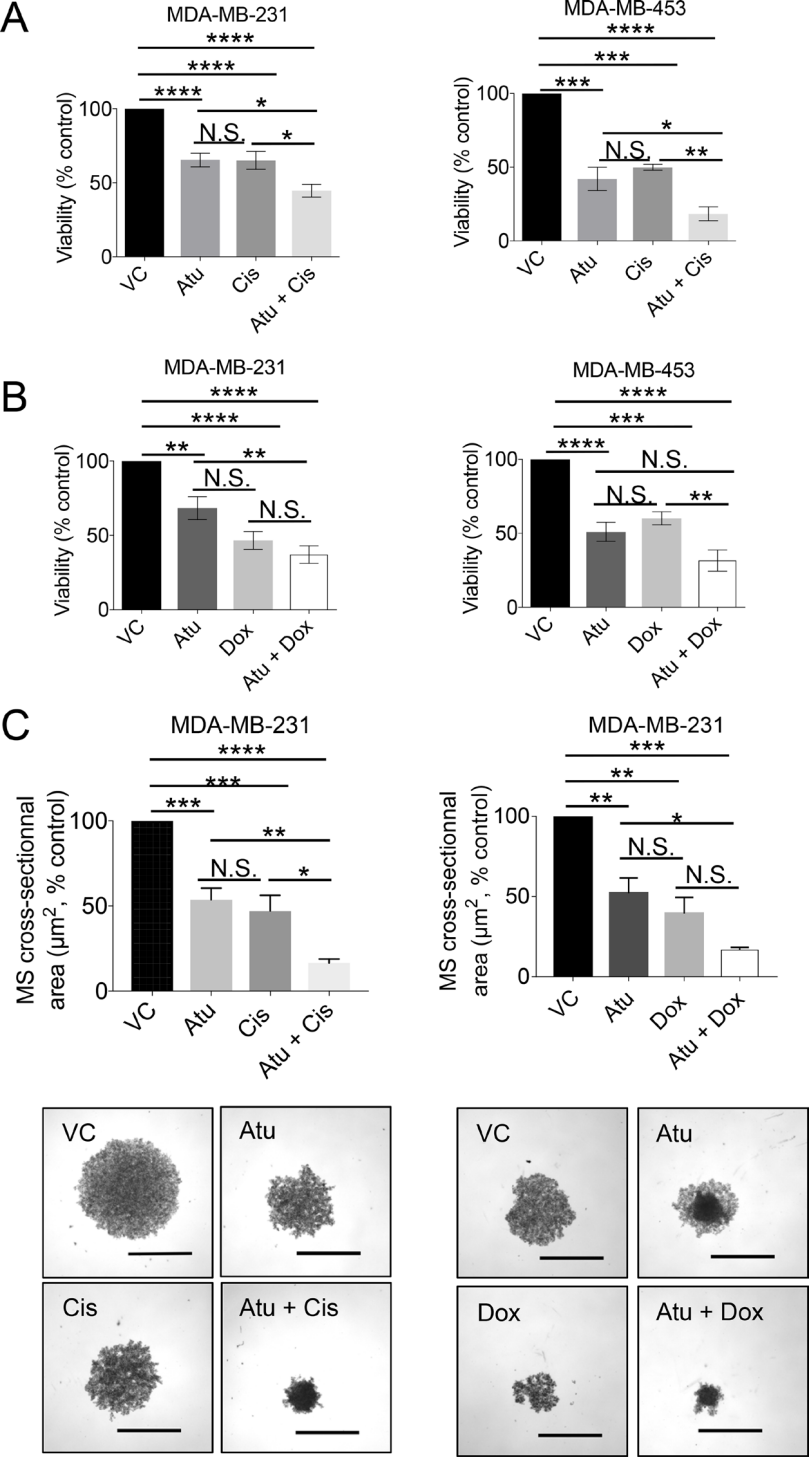
Assessment of cell viability upon atuveciclib treatment in combination with chemotherapy in MDA-MB-231 or MDA-MB453 (**A**) MDA-MB-231 (left panel) or MDA-MB-453 (right panel) cells were seeded into 96-well plates. After 24 hours, cells were treated with atuveciclib in combination with cisplatin for 4 days. Cell viability was assessed using the WST-1 proliferation reagent. Data are presented as the percentages of vehicle control (VC) treated cells. Results represent the means ± SEM of six (MDA-MB-231) or three (MDA-MB-453) independent experiments. ^*^P < 0.05; ^**^P < 0.01; ^***^P < 0.001, ^****^P < 0.0001. (**B**) Similar experiment as in A using a combination of atuveciclib and doxorubicin for 4 days. Data are presented as the percentages of vehicle control (VC) treated cells. Results represent the means ± SEM of six (MDA-MB-231) or three (MDA-MB-453) independent experiments. ^**^P < 0.01; ^***^P < 0.001, ^****^P < 0.0001. N.S. stands for non-significant. (**C**) MDA-MB-231 mammospheres (MS) were grown in 96-well low attachment plates in the presence of atuveciclib in combination with cisplatin (left panels) or doxorubicin (right panels). After 8 days, mammospheres were imaged to determine cross-sectional area. Data are presented as the percentages of VC-treated cells. Results represent the means ± SEM of three independent experiments. Representative images are depicted in the lower panels. ^*^P < 0.05; ^**^P < 0.01; ^***^P < 0.001, ^****^P < 0.0001. N.S. stands for non-significant. Scale bar represents 1000 μm.

These results led to test whether CDK9 inhibition might increase TNBC cell sensitivity to conventional chemotherapeutic agents commonly used for treating TNBC such as cisplatin or doxorubicin [40]. To this end, we treated high-*CDK9* TNBC cell lines (MDA-MB-231 and MDA-MB-453) with atuveciclib in combination with cisplatin or doxorubicin and subjected cells to cell viability assays. Atuveciclib in combination with cisplatin significantly inhibited cell viability (Figure 6A); and a similar trend was observed when atuveciclib was combined with doxorubicin (Figure 6B). Moreover, co-treatment of MDA-MB-231 mammospheres with atuveciclib and cisplatin resulted in significantly reduced mammosphere size (Figure 6C, left panels). A similar trend was observed when atuveciclib was combined with doxorubicin (Figure 6C, right panels).

## DISCUSSION

TNBC accounts for approximately 20% of all breast cancers [1, 2]. So far, no molecularly targeted therapy has been FDA-approved to treat patients with TNBC primarily due to lack of validated therapeutic targets. The current standard of care for patients with TNBC is limited to the combination of conventional cytotoxic chemotherapy in both primary and metastatic settings, radiation and surgery in early disease [1, 2]. TNBC patients receiving the current standard of care tend to experience early tumor recurrence and metastatic progression of the disease. Thus, there are significant unmet clinical needs in treating patients with TNBC and this has led to a considerable amount of interest in establishing targeted therapies for these patients [5, 6].

CDKs have long been considered promising anti-cancer targets because of their essential roles in cancer cell cycle progression. Despite a few decades of intensive studies on CDKs, pan-CDK inhibitors offer a rather limited therapeutic window due to adverse effects, a limited combination with other cytotoxic agents and thus, few investigations in TNBC [18–24]. Therefore, it became critical to elucidate and dissect the precise role of different CDKs in various malignancies, in order to explore specific single-CDK targeting agents therapeutically. Recently, selective CDK4/6 inhibitors in combination with aromatase inhibitors or fulvestrant were FDA-approved for hormone receptor-positive, HER2-negative advanced or metastatic breast cancer (e.g. palbociclib, ribocilcib, abemaciclib) [18–24]. Interestingly, abemaciclib differs from the other CDKs inhibitors because it specifically targets CDK4/6 and exerts some inhibitory effects on CDK9 [41]. Moreover, abemaciclib is the only inhibitor in this class with activity as a single agent in breast cancer and other solid tumors, suggesting a possible involvement of CDK9 inhibition [42, 43].

The data presented in this manuscript, establishing that *CDK9* expression is associated with poor prognosis among patients with TNBC and that high-*CDK9* TNBC cells are sensitive to CDK9 inhibition raising the possibility of specific CDK9 targeting as a unique approach in the treatment of aggressive forms of TNBC. Notably, CDK9 is not directly involved in cell cycle progression. It belongs to a class of non-cell cycle CDKs known as transcriptional CDKs. CDK9 dysregulation has been described in several human malignancies including tumors of the breast [33, 44], prostate [45], and lung [46] origins. CDK9 interacts with cyclin T1 to form the P-TEFb complex, which regulates transcription of pro-tumorigenic genes such as *MYC*, *MCL1*, and Hexamethylene Bisacetamide Inducible 1 (*HEXIM1*) [25, 26, 33, 47]. Of these CDK9 targets, the MYC oncoprotein is of particular interest as it was found to be dysregulated in approximately 50% of TNBC tumors, and aberrant MYC pathway activation is associated with poor clinical outcomes [27]. Despite three decades of efforts directed toward developing direct inhibitors of oncogenic MYC transcriptional activity for clinical use, MYC has remained “undruggable” [28, 48]. Here we show that selective CDK9 inhibition results in downregulation of MYC. As MYC is a transcriptional target of CDK9-reguated P-TEFb complex [25, 26, 33], this suggests that CDK9 inhibition might be particularly promising in MYC-driven TNBC.

Using atuveciclib, a small molecule that selectively targets CDK9, we found that CDK9 targeting reduced cell proliferation and triggered apoptosis in high-*CDK9* TNBC cells. These antitumor effects appear to reflect CDK9 inhibition, as demonstrated by decreased phosphorylation of RNA pol II at Ser2, and reduced protein expression levels of MYC and MCL1. It should be noted that in breast cancer, a small population of tumor cells known as cancer stem-like cells (CSLCs) are equipped with progenitor-like ability to confer resistance to the current standard chemotherapy and contribute to disease progression. The present study provides evidence that atuveciclib suppresses the invasive cellular phenotype of high-*CDK9* TNBC cells and disrupts the activities of CSLCs in 3D mammospheres, a cell population with high cellular demand for CDK9 activity. TNBC are aggressive tumors with the ability to develop metastases in visceral organs and resistance to therapies. Preclinical studies showed that these tumors are enriched in CSLCs responsible for the clinical behavior. Hence, agents affecting CSLCs can be effective in TNBC. These observations on the effects of a small molecule CDK9 inhibition on CSLCs are important new insights in TNBC, and are consistent with CDK9 loss-of-function phenotypes observed in glioblastoma stem cells [49]. Key roles in therapy resistance have been attributed to MCL1 and MYC. First, *MCL1* and *MYC* were found to be co-amplified in residual TNBC tumors after neoadjuvant chemotherapy [50]. Second, MCL1 and MYC have recently been involved in maintaining CSLCs resistance to chemotherapy in TNBC [51]. In this study, the selective CDK9 inhibitor atuveciclib reduced expression of MCL1 and MYC, blocked mammosphere growth in 3D and exhibited potent antineoplastic effects. Finally, we found that CDK9 inhibition by atuveciclib increased sensitivity of TNBC cells to cytotoxic chemotherapeutic agents. This suggests that CDK9 inhibition may be effective in TNBC and in particular in combination with the standard of care in aggressive forms of *CDK9*-driven TNBC. Additional studies using relevant mouse models to assess the effects of atuveciclib *in vivo* would be important and may have important clinical-translational implications for the treatment of aggressive forms of CDK9-high expressing TNBC. Taken together, our study warrants further preclinical animal drug efficacy studies and early phase clinical evaluation on the potential clinical utility of small molecule CDK9 inhibitors against TNBC.

## MATERIALS AND METHODS

### Reagents

Atuveciclib (BAY1143572) was purchased from ActiveBiochem. Doxorubicin hydrochloride and cisplatin were purchased from Sigma-Aldrich (#D1515-10MG, #1134357-100MG). Dimethyl sulfoxide (DMSO) served as vehicle control (VC) for all drugs for *in vitro* experiments.

### Human breast cancer cell lines

Cell lines were purchased from ATCC. MDA-MB-231, MDA-MB-453, MDA-MB-436, MDA-MB-157, HBL100 were grown in DMEM supplemented with 10% fetal bovine serum (FBS) and penicillin (100 units/mL)/streptomycin (100 ng/mL). HCC1937, HCC3153, BT549 were grown in RPMI 1640 medium with 10% FBS and penicillin (100 units/mL)/streptomycin (100 ng/mL).

### Cell viability assays

WST-1 assays were used according to the manufacturer’s (Roche) instructions to evaluate cell viability. Briefly, 2,000 cells per well were seeded into 96-well plates in the presence of atuveciclib (0.01 or 0.03 or 0.1 or 0.3 or 1 or 3 or 10 or 30 µM). Three (BT549, MDA-MB-436, HCC3153 and MDA-MB-157) or four (MDA-MB-453, MDA-MB-231, HCC1937 and HBL100) independent experiments were each performed in triplicates. For combinatorial approaches, atuveciclib (1 µM) was tested with cisplatin (5 µM) or doxorubicin (0.1 µM). After four days at 37°C in 5% CO2, 10% (v/v) WST-1 reagent was added and absorbance was measured at 450 nm and 600 nm using a Synergy HT plate reader. Viability was analyzed using the Gen5 software (BioTek).

### *In vitro* clonogenic assays

Three-dimensional laminin-rich extracellular matrix (3D lrECM) on-top cultures were performed as described [37]. Briefly, cells were seeded into 96-well plates on a thin layer of Engelbreth-Holm-Swarm (EHS) tumor extract (growth factor-reduced Matrigel) (Corning) at a density of 2,000 cells/well in propagating medium plus 1% FBS. After 24 hours, cells were treated with atuveciclib (0.5, 1 or 3 µM) and colonies were imaged after four days. Colonies were quantified using Cytation 3 and the Gen5 software (BioTek). Only colonies with a diameter equal or greater than 60 µm were analyzed.

### Invasion assays

Cultrex^®^ Cell Invasion Assay (Trevigen) was used according to the manufacturer’s instructions. Briefly, cells were resuspended in 1X spheroid formation ECM (extra cellular matrix) and seeded at 5,000 cells/well into 96-well ultra-low attachment round bottom plates. Cells were incubated for 72 hours to promote sphere formation before adding invasion matrix to each well. Subsequently, medium containing atuveciclib (1 or 3 µM) was added to each well. After six days, spheres were imaged using a Cytation 3 (BioTek) plate reader. ImageJ software (Version 1.50i, NIH) was used to determine the cross-sectional area (µm^2^) of the invasive spheres.

### Mammosphere assays

Mammospheres from MDA-MB-231 and MDA-MB-453 cell lines were generated using MammoCult™ Human Medium Kit (Stem Cell Technologies) according to the manufacturer’s recommendations. Briefly, adherent cells were dissociated and seeded into 6-well ultra-low attachment plates in complete Mammocult™ media (MDA-MB-231: 2 × 10^4^ cells/well, MDA-MB-453: 4 × 10^4^ cells/well). After seven days, mammospheres were dissociated with Trypsin and 2,000 cells/well were seeded into 96-well ultra-low attachment round bottom plates and treated in triplicates with atuveciclib (1 or 3 µM). For combinatorial approaches, atuveciclib (1 µM) was tested with cisplatin (5 µM) or doxorubicin (0.1 µM). After seven days, mammospheres were imaged using Cytation 3 and Gen5 software (BioTek) and cross-sectional area (in µm^2^) was assessed using ImageJ software (Version 1.50i, NIH).

### Aldehyde dehydrogenase (ALDH) activity

ALDH activity was measured after four days of atuveciclib treatment (0.5 or 1 or 3 µM) using the ALDEFLUOR™ kit (Stem Cell Technologies) according to the manufacturer’s instruction. Briefly, 5 × 10^5^ cells per experimental condition were incubated with 5 µL of activated ALDEFLUOR™ with or without Diethylaminobenzaldehyde (DEAB), an ALDH1 inhibitor. After incubation for 45 min at 37°C cells were analyzed by flow cytometry (BD LSRFortessa Analyzer cytometer).

### Apoptosis assay

For analysis of apoptosis, the BD Pharmingen FITC Annexin V Apoptosis Detection Kit I (BD Biosciences) was used according to the manufacturer’s instructions. Briefly, MDA-MB-231, MDA-MB-453 and HCC1937 cells were seeded into 6-well plates. The following day, cells were incubated with atuveciclib (0.5 or 1 or 3 µM) for four days. Following treatment, cells were harvested using trypsin, washed three times with PBS, and stained with Propidium Iodide Staining Solution (PI) and FITC Annexin V. Stained samples were analyzed by flow cytometry (BD LSRFortessa Analyzer cytometer) and FlowJo software (version 10.3). Percentage of apoptotic cells (Annexin-V positive and Annexin-V+PI double positive) was quantified.

### Flow cytometry

To analyze the CD24^low^/CD44^high^ population, 5 x 10^5^ cells treated with atuveciclib (0.5 or 1 or 3 µM) for 4 days were resuspended in PBS supplemented with 2% FBS, 10 mM EDTA mixed with a mouse anti-CD24-A647 (Biolegend 311109) and anti-CD44-FITC (Biolegend 338803) (5 µL/100µL/test) or alternatively with the isotypes control mouse FITC-IgG1 (Biolegend 400107) and mouse IgG2a- A647 (Biolegend 400234). After incubation for 45 min at 4°C, cells were washed three times in PBS supplemented with 2% FBS and 10 mM EDTA. Cells were stained with DAPI to exclude dead cells. At least 10,000 events were collected for each sample (BD LSRFortessa Analyzer cytometer) and data were analyzed using FlowJo software (Version 10.3).

### Western blot analysis

Cell pellets were lysed in phosphorylation lysis buffer (50 mmol/L Hepes, 150 mmol/L NaCl, 1 mmol/L MgCl_2_, 0.5% Triton, 10% glycerol, 0.5% sodium deoxycholate, pH 7.9) supplemented with phosphatase and protease inhibitors. Protein concentrations were determined by Bradford assay (Bio-Rad) using the Synergy HT plate reader and Gen5 software (BioTek Instruments). Equal amounts of total protein lysates were separated by SDS-PAGE (Bio-Rad) and transferred to Immobilon PVDF membranes (Millipore). Membranes were blocked with 5% milk- 1% BSA in 1× TBST and incubated with primary antibodies overnight at 4°C. β-actin and HSP90 antibodies were from Santa Cruz Biotechnology, all other primary antibodies were obtained from Cell Signaling Technology and used at a dilution of 1:1,000. Following primary antibody incubation, membranes were washed three times with 1X TBST and incubated with anti-rabbit (GE Healthcare) or anti-mouse (Bio-Rad) horseradish peroxidase (HRP)-conjugated secondary antibodies for 1 hour. Membranes were then washed three times with 1X TBST and developed with WesternBright ECL HRP substrate (Advansta) and autoradiography film (Denville Scientific) or ChemiDoc Imaging System (BioRad). Antibody information is provided in Supplementary Table 1.

### Bioinformatics analyses

RNA-seq data along with clinical information available for 89 TNBC patients were retrieved from The Cancer Genome Atlas (TCGA) (list of the sample IDs provided in Supplementary Table 2). The cutoff finder software program [52] was used to determine an optimized cutoff to create high- versus low-*CDK9* patient groups. Prism (Version7) from GraphPad Software was used to generate a Kaplan-Meier curve to investigate prognostic significance of *CDK9* expression. *CDK9* expression data from breast cancer cell lines were retrieved from University of California, Santa Cruz (UCSC) Xena browser (https://xenabrowser.net/) to determine *CDK9* expression (Log2 expression) in a panel of TNBC cell lines (Neve_2006 dataset) [31]. A cancer prognostic database (PROGgeneV2 database) [53] was used to generate Kaplan-Meier curves to study associations between *CDK9* expression and recurrence-free survival (high: *n* = 77 or low: *n* = 77) (GSE9893 dataset) [30] and metastasis-free survival (MFS) (high: *n* = 148 patients; or low: *n* = 147 patients) (NKI dataset) [38] in breast cancer patients.

### Statistical analysis

All results are presented as mean +/– standard error of the mean (S.E.M.). Unless otherwise indicated, all statistical analyses were performed based on one-way analysis of variance (ANOVA) using Prism (Version7) from GraphPad Software. Significant treatment effects were subsequently delineated by using Dunnett’s post hoc test for increasing atuveciclib concentrations or Tukey’s post hoc test for drug combinations. The stratification of TNBC patients based on *CDK9* expression was performed using Mantel-Cox test. *P* values <0.05 were assumed to indicate statistical significance throughout the study. Spearman correlation was employed to assess the correlation between CDK9 mRNA expression and sensitivity to atuveciclib in TNBC cell lines.

## SUPPLEMENTARY MATERIALS FIGURES AND TABLES



## Abbreviations

ALDH1: aldehyde dehydrogenase
BCSLC: breast cancer stem-like cell
CDK: cyclin-dependent kinase
CLCS: cancer stem-like cell
ER: estrogen receptor
HER2: human epidermal growth factor 2
PDX: patient-derived tumor xenograft
PR: progesterone receptor
P-TEFb: positive transcription elongation factor b
TNBC: triple-negative breast cancer
