# Supplementary material for: Antineoplastic effects of selective CDK9 inhibition with atuveciclib on cancer stem-like cells in triple-negative breast cancer
Source: Oncotarget. 2018 Dec 18;9(99):37305–18. doi: 10.18632/oncotarget.26468 (PMC6324664; doi:10.18632/oncotarget.26468)
Supplement: Supplementary file 1 [file oncotarget-09-37305-s001.pdf]

Antineoplastic effects of selective CDK9 inhibition with atuveciclib on cancer stem-like cells in triple-negative breast cancer

SUPPLEMENTARY MATERIALS

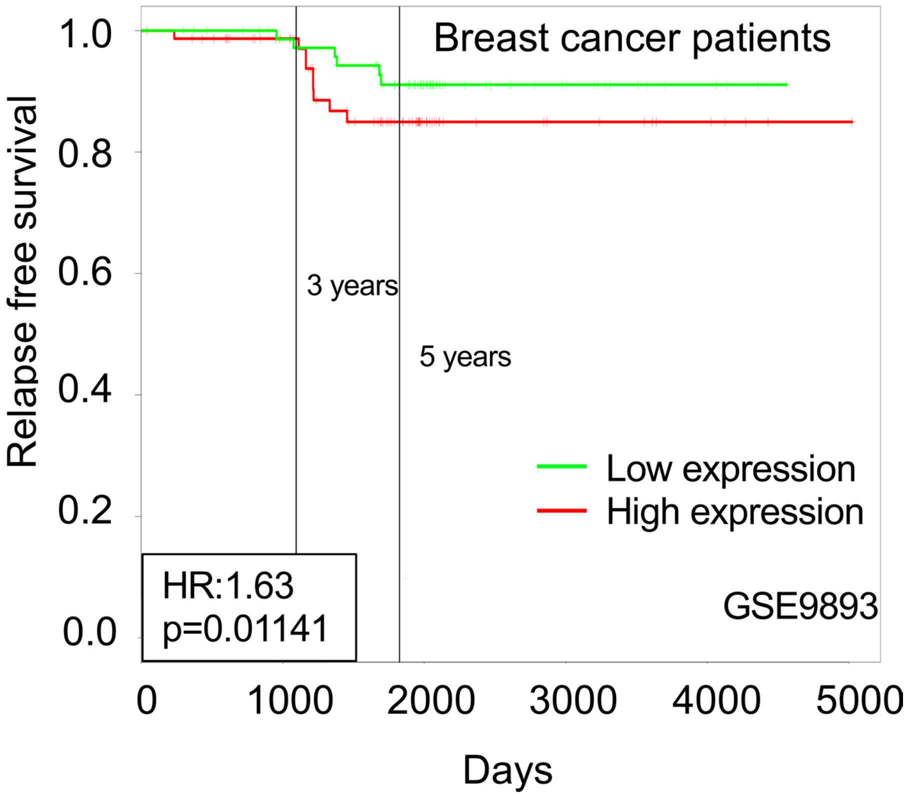

Supplementary Figure 1: *CDK9* expression and clinical breast cancer outcome. Kaplan-Meier curve indicating the correlation between CDK9 expression (high: n = 77, low: n = 77) and relapse-free survival (RFS) in breast cancer patients. Prognostic Plot created with PROGgeneV2 software using the GSE9893 dataset. The cohort was divided at median of gene expression. Log-rank (Mantel-Cox) test P = 0.01141, Hazard Ratio (HR): 1.63.

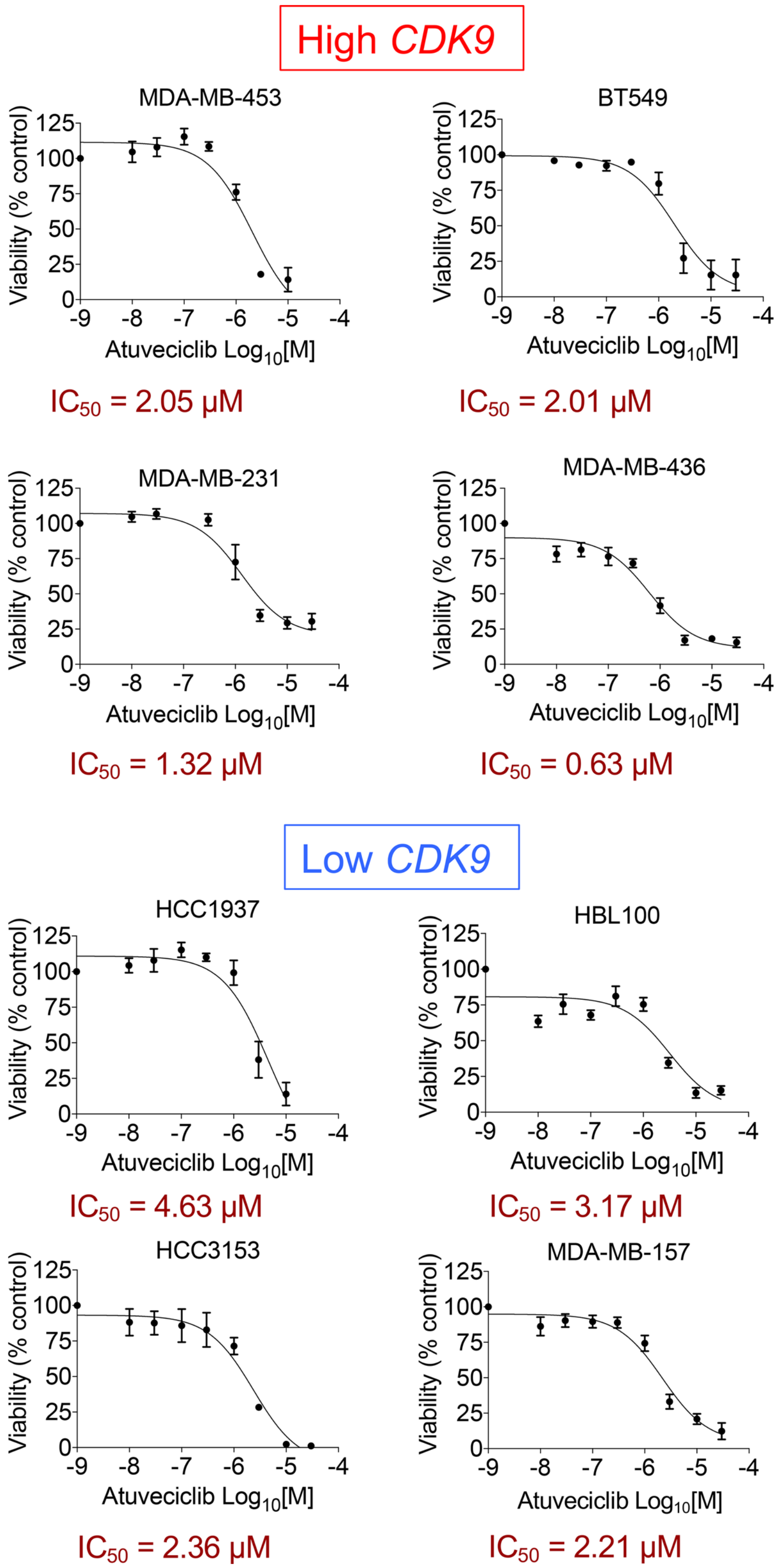

Supplementary Figure 2: Effect of atuveciclib on cell viability of TNBC cell lines exhibiting high (upper panels) or low (lower panels) *CDK9* expression. Upper panels display TNBC cell lines expressing high *CDK9* (MDA-MB-453, BT549, MDA-MB-231, MDA-MB-436) and the lower panels display TNBC cell lines expressing low *CDK9* (HCC1937, HBL100, HCC3153, MDA-MB-157). Cells were seeded in 96-well plates and treated with increasing doses of atuveciclib for 4 days. WST-1 cell proliferation reagent was used to assess cell viability. Data are presented as the percentages of vehicle control (VC) treated cells. Results represent the means ± SEM of three (BT549, MDA-MB-436, HCC3153 and MDA-MB-157) or four (MDA-MB-453, MDA-MB-231, HCC1937 and HBL100) independent experiments, each performed in triplicates.

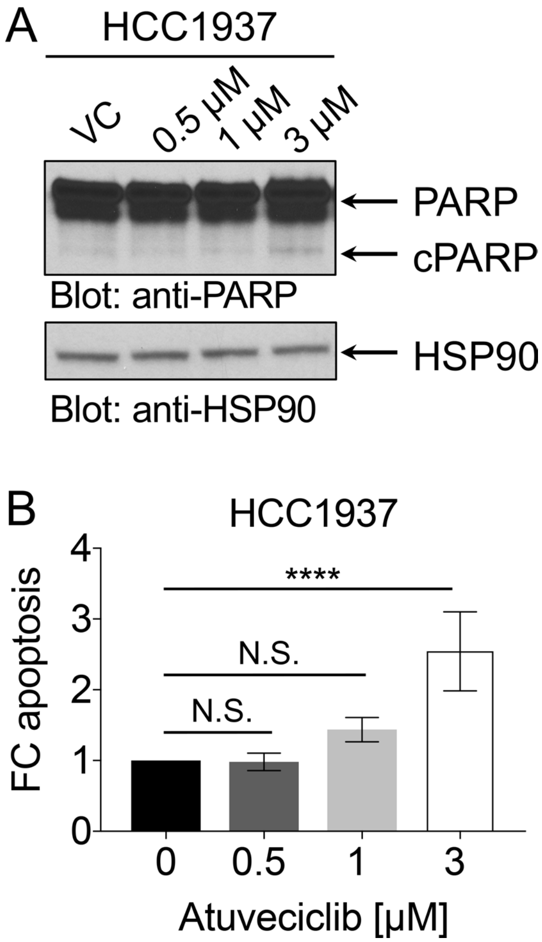

Supplementary Figure 3: Effect of atuveciclib on apoptosis in a low *CDK9* expressing TNBC cell line HCC1937. (**A**) HCC1937 cells were treated with atuveciclib at indicated concentrations for 24 hours. Equal amounts of lysates were analyzed by SDS-PAGE and immunoblotted for antibodies against PARP (which detects both cleaved (cPARP) and full length PARP) and HSP90. (**B**) HCC1937 cells were treated with atuveciclib at indicated concentrations for 4 days. Cells were stained with Annexin-V and propidium Iodide (PI) antibodies and then analyzed by flow cytometry. The sum of Annexin-V positive and Annexin-V+PI double positive cells was used to determine the amount of total apoptotic cells. Data are presented as the fold change (FC) over vehicle control (VC) treated cells. Results represent the means ± SEM of six independent experiments. $^{****}P < 0.0001$. N.S. stands for non-significant.

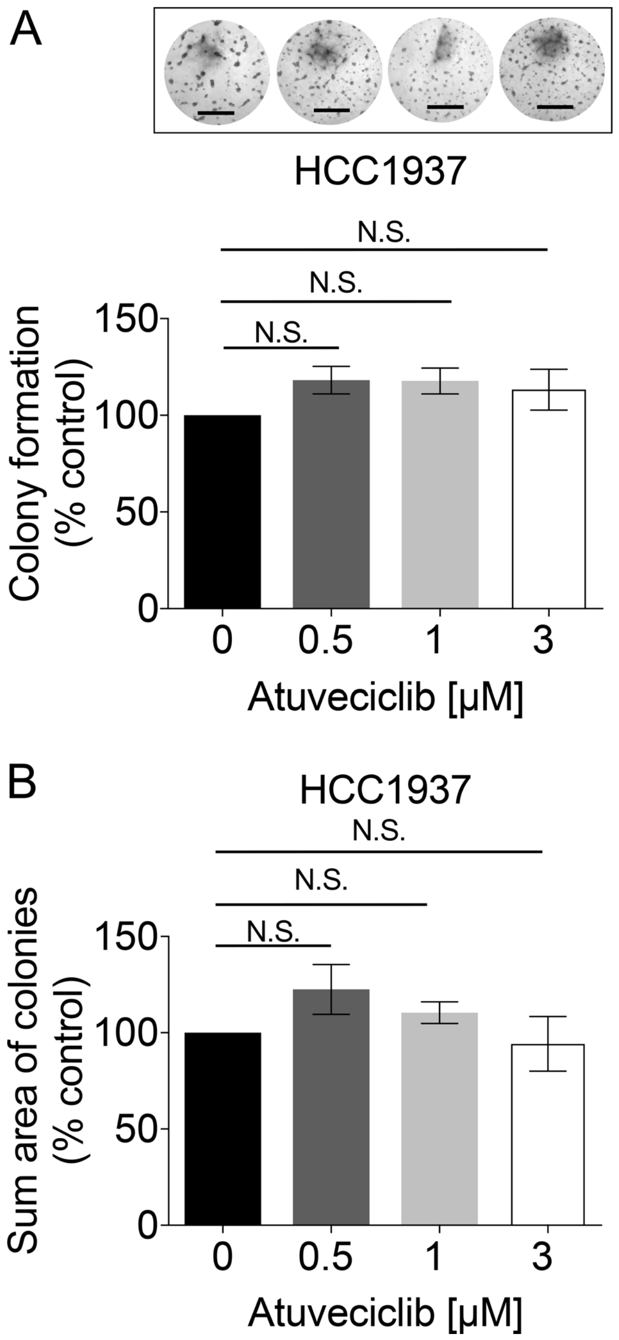

Supplementary Figure 4: Effect of increasing doses of atuveciclib on colony formation in a low-CDK9 expression TNBC cell line. HCC1937 cells were seeded into 96-well plates on a thin layer of EHS tumor extract. After 24 hours, cells were treated with atuveciclib at indicated concentrations. After 6 days plates were imaged, colonies with a diameter ≥ 60 µm were scored positive. (**A**) Data are presented as the percentages of vehicle control (VC) treated cells. Results represent the means ± SEM of four independent experiments. Representative images are depicted in the upper panel. Scale bar 1000 µm. N.S. stands for non-significant. (**B**) Experiment as in A. The cross-sectional area of colonies with a diameter ≥ 60 µm was measured and summed up. Sum cross-sectional area data are presented as the percentage of vehicle control (VC) treated cells. Results represent the means ± SEM of four independent experiments. N.S. stands for non-significant.

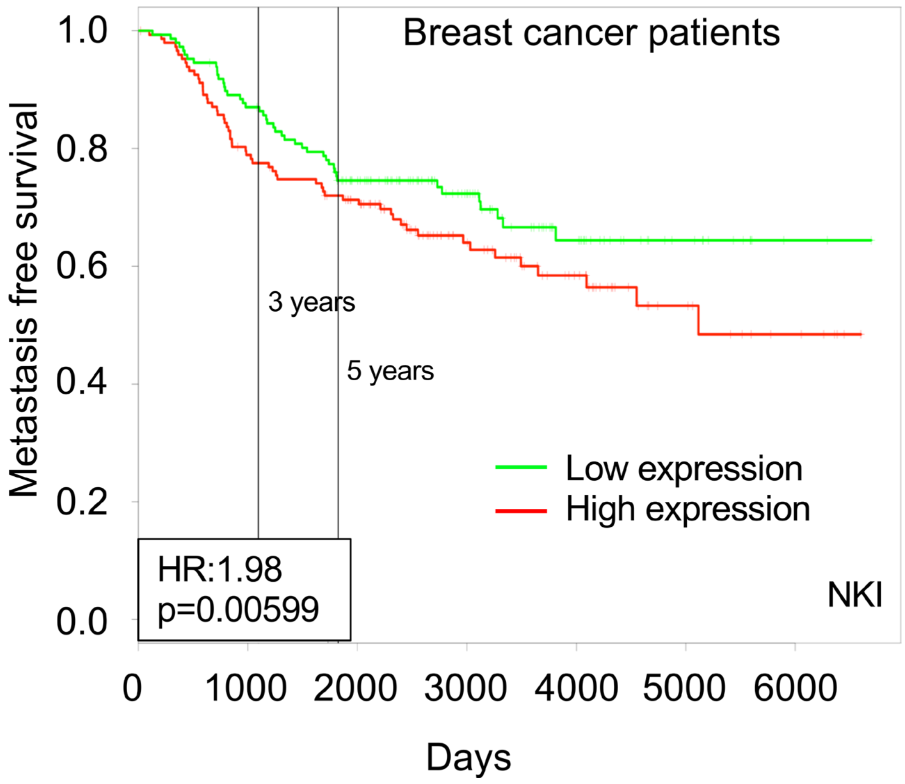

Supplementary Figure 5: *CDK9* expression and clinical breast cancer outcome. Kaplan-Meier curve indicating the correlation between CDK9 expression (high: $n = 148$, low: $n = 147$) and metastasis-free survival (MFS) in breast cancer patients. Prognostic Plot was created with PROGgeneV2 software using the NKI dataset. The cohort was divided at median of gene expression. Log-rank (Mantel-Cox) test $P = 0.00599$, Hazard Ratio (HR): 1.98.

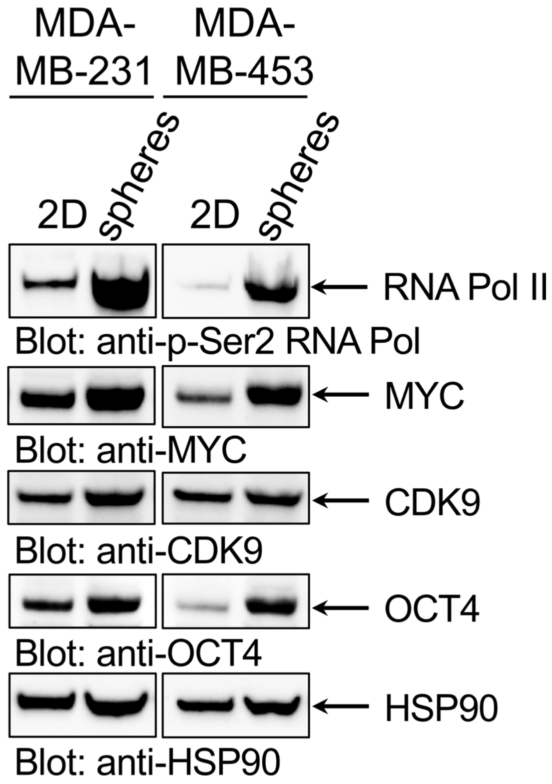

Supplementary Figure 6: Comparison of protein levels in 2D cell lines and 3D mammospheres from TNBC cell lines. MDA-MB-231 (left panels) and MDA-MB-453 (right panels) cells (2D) or mammospheres (spheres) were lysed and subjected to SDS-PAGE. Same amounts of lysates were used for analysis and immunoblotted for antibodies against RNA Pol II pSer2, MYC, CDK9, OCT4 and HSP90. ChemiDoc MP Imaging System (BioRad) was used for visualization.

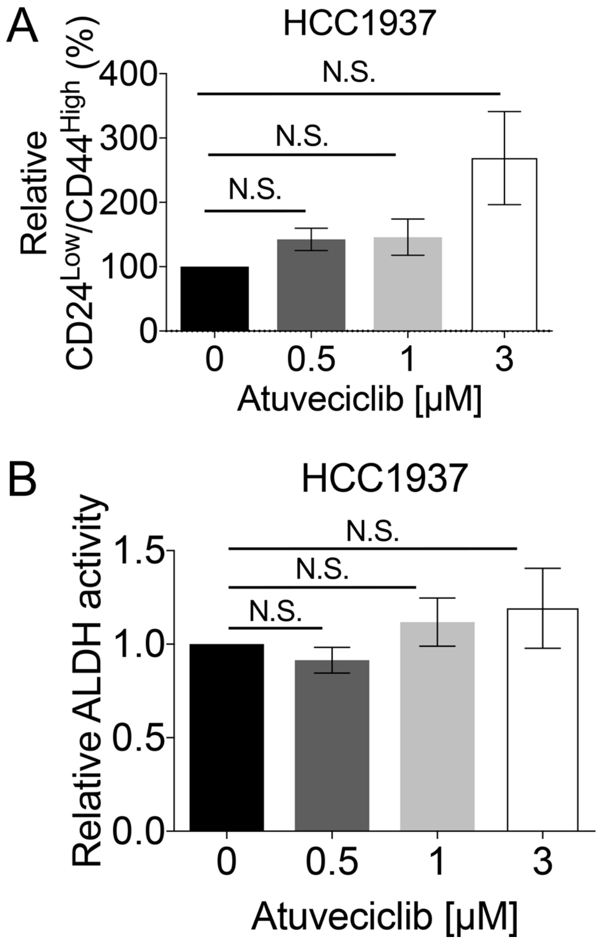

Supplementary Figure 7: Effect of atuveciclib on stem-like cancer cells from a low-CDK9 expression TNBC cell line. (**A**) HCC1937 cells were seeded into 6-well plates and treated with atuveciclib at indicated concentrations. After 4 days, cells were stained with anti-CD24 and anti-CD44 antibodies and analyzed by flow cytometry. Data are presented as the percentages of VC-treated cells. Results represent the means ± SEM of four independent experiments. N.S. stands for non-significant. (**B**) Cells were submitted to the same treatment as in A. After 4 days, cells were stained with ALDEFLUOR with or without DEAB and were analyzed by flow cytometry. Data are presented as the fold-change over vehicle control (VC) treated cells. Results represent the means ± SEM of six independent experiments. N.S. stands for non-significant.

Supplementary Table 1: List of antibodies used in the study

Antibody	Clone	Company	Catalog number	Specie
PARP	46D11	Cell signaling	9532	Rabbit monoclonal
CDK9	CD12F7	Cell signaling	2316	Rabbit monoclonal
MCL1		Cell signaling	4572	Rabbit polyclonal
RpB1 CTD	4H8	Cell signaling	2629	Mouse monoclonal
phospho-Rpb1 (Ser2) CTD	E1Z3G	Cell signaling	13499	Rabbit monoclonal
phospho-Rpb1 (Ser5) CTD	D9N5I	Cell signaling	13523	Rabbit monoclonal
OCT4		Cell signaling	2750	Rabbit polyclonal
HSP90 alpha/beta	H-114	Santa cruz	sc-7947	Rabbit polyclonal
MYC	Y69	Abcam	32072	Rabbit monoclonal
Donkey anti-rabbit		GE Healthcare		
Goat anti-mouse		Bio-Rad	1706516	

Supplementary Table 2: List of the TNBC cancer sample ID retrieved from the TCGA

TCGA breast cancer sample ID
TCGA-A1-A0SK-01
TCGA-A1-A0SO-01
TCGA-A1-A0SP-01
TCGA-A2-A04P-01
TCGA-A2-A04Q-01
TCGA-A2-A04T-01
TCGA-A2-A04U-01
TCGA-A2-A0CM-01
TCGA-A2-A0D0-01
TCGA-A2-A0D2-01
TCGA-A2-A0ST-01
TCGA-A2-A0SX-01
TCGA-A2-A0T0-01
TCGA-A2-A0T2-01
TCGA-A2-A0YE-01
TCGA-A2-A0YM-01
TCGA-A7-A0CE-01
TCGA-A7-A0DA-01
TCGA-A8-A07C-01
TCGA-A8-A07O-01
TCGA-A8-A08R-01
TCGA-A8-A09X-01
TCGA-AN-A04D-01
TCGA-AN-A0AL-01
TCGA-AN-A0AR-01
TCGA-AN-A0AT-01
TCGA-AN-A0FL-01
TCGA-AN-A0FX-01
TCGA-AN-A0G0-01
TCGA-AN-A0XU-01
TCGA-AO-A03U-01
TCGA-AO-A0J2-01
TCGA-AO-A0J4-01
TCGA-AO-A0J6-01
TCGA-AO-A0JL-01
TCGA-AO-A124-01
TCGA-AO-A128-01
TCGA-AO-A129-01
TCGA-AO-A12F-01
TCGA-AQ-A04J-01
TCGA-AR-A0TS-01
TCGA-AR-A0TU-01
TCGA-AR-A0U0-01
TCGA-AR-A0U1-01

TCGA-AR-A0U4-01
TCGA-AR-A1AI-01
TCGA-AR-A1AQ-01
TCGA-AR-A1AR-01
TCGA-AR-A1AY-01
TCGA-B6-A0IE-01
TCGA-B6-A0IK-01
TCGA-B6-A0IQ-01
TCGA-B6-A0RE-01
TCGA-B6-A0RG-01
TCGA-B6-A0RN-01
TCGA-B6-A0RS-01
TCGA-B6-A0RT-01
TCGA-B6-A0RU-01
TCGA-B6-A0WX-01
TCGA-BH-A0AV-01
TCGA-BH-A0B3-01
TCGA-BH-A0B9-01
TCGA-BH-A0BG-01
TCGA-BH-A0BL-01
TCGA-BH-A0BW-01
TCGA-BH-A0E0-01
TCGA-BH-A0E6-01
TCGA-BH-A0RX-01
TCGA-BH-A0WA-01
TCGA-BH-A18G-01
TCGA-BH-A18Q-01
TCGA-BH-A18T-01
TCGA-BH-A18V-01
TCGA-BH-A1EW-01
TCGA-C8-A12V-01
TCGA-C8-A131-01
TCGA-C8-A134-01
TCGA-D8-A13Z-01
TCGA-D8-A142-01
TCGA-D8-A143-01
TCGA-D8-A147-01
TCGA-E2-A14N-01
TCGA-E2-A14R-01
TCGA-E2-A14X-01
TCGA-E2-A150-01
TCGA-E2-A158-01
TCGA-E2-A159-01
TCGA-E2-A1AZ-01
TCGA-E2-A1B6-01
